# Matrix stiffness-upregulated LOXL2 promotes fibronectin production, MMP9 and CXCL12 expression and BMDCs recruitment to assist pre-metastatic niche formation

**DOI:** 10.1186/s13046-018-0761-z

**Published:** 2018-05-04

**Authors:** Sifan Wu, Qiongdan Zheng, Xiaoxia Xing, Yinying Dong, Yaohui Wang, Yang You, Rongxin Chen, Chao Hu, Jie Chen, Dongmei Gao, Yan Zhao, Zhiming Wang, Tongchun Xue, Zhenggang Ren, Jiefeng Cui

**Affiliations:** 1 Liver Cancer Institute, Zhongshan Hospital, Fudan University and Key Laboratory of Carcinogenesis and Cancer Invasion, Ministry of Education, 136 Yi Xue Yuan Road, Shanghai, 200032 People’s Republic of China; 2 Department of Radiology, Shanghai Cancer Center, Fudan University, Shanghai, 200032 People’s Republic of China; 3 Department of Urology, Zhongshan Hospital, Fudan University, Shanghai, 200032 People’s Republic of China; 4 Department of Oncology, Zhongshan Hospital, Fudan University, Shanghai, 200032 People’s Republic of China

**Keywords:** Hepatocellular carcinoma, Matrix stiffness, LOXL2, Pre-metastatic niche

## Abstract

**Background:**

Higher matrix stiffness affects biological behavior of tumor cells, regulates tumor-associated gene/miRNA expression and stemness characteristic, and contributes to tumor invasion and metastasis. However, the linkage between higher matrix stiffness and pre-metastatic niche in hepatocellular carcinoma (HCC) is still largely unknown.

**Methods:**

We comparatively analyzed the expressions of LOX family members in HCC cells grown on different stiffness substrates, and speculated that the secreted LOXL2 may mediate the linkage between higher matrix stiffness and pre-metastatic niche. Subsequently, we investigated the underlying molecular mechanism by which matrix stiffness induced LOXL2 expression in HCC cells, and explored the effects of LOXL2 on pre-metastatic niche formation, such as BMCs recruitment, fibronectin production, MMPs and CXCL12 expression, cell adhesion, etc.

**Results:**

Higher matrix stiffness significantly upregulated LOXL2 expression in HCC cells, and activated JNK/c-JUN signaling pathway. Knockdown of integrin β1 and α5 suppressed LOXL2 expression and reversed the activation of above signaling pathway. Additionally, JNK inhibitor attenuated the expressions of p-JNK, p-c-JUN, c-JUN and LOXL2, and shRNA-c-JUN also decreased LOXL2 expression. CM-LV-LOXL2-OE and rhLOXL2 upregulated MMP9 expression and fibronectin production obviously in lung fibroblasts. Moreover, activation of Akt pathway contributed to LOXL2-induced fibronectin upregulation. LOXL2 in CM as chemoattractant increased motility and invasion of BMCs, implicating a significant role of LOXL2 in BMCs recruitment. Except that, CM-LV-LOXL2-OE as chemoattractant also increased the number of migrated HCC cells, and improved chemokine CXCL12 expression in lung fibroblasts. The number of HCC cells adhered to surface of lung fibroblasts treated with CM-LV-LOXL2-OE was remarkably higher than that of the control cells. These results indicated that the secreted LOXL2 facilitated the motility of HCC cells and strengthened CTCs settlement on the remodeled matrix “soil”.

**Conclusion:**

Integrin β1/α5/JNK/c-JUN signaling pathway participates in higher matrix stiffness-induced LOXL2 upregulation in HCC cells. The secreted LOXL2 promotes fibronectin production, MMP9 and CXCL12 expression and BMDCs recruitment to assist pre-metastatic niche formation.

**Electronic supplementary material:**

The online version of this article (10.1186/s13046-018-0761-z) contains supplementary material, which is available to authorized users.

## Background

Increasing evidences suggest that matrix stiffness influences biological behaviors of cells such as cell proliferation [1], differentiation [2, 3], migration [4], and metabolism [5], regulates disease-associated genes/miRNA expression [6–9], stemness [10], chemoresistance [11], and contributes to tumor invasion and metastasis [12]. Hepatocellular carcinoma (HCC) is one of the most frequent tumor in China and the third leading cause of cancer-related mortality worldwide [13]. Over 80% of HCC patients have cirrhosis or advanced fibrosis background. The mortality rate of HCC with cirrhosis background rises in some developed countries [14]. Currently, higher liver stiffness has become a strong predictor in clinic for HCC development and prognosis [1, 15]. Our previous studies have demonstrated that increased matrix stiffness not only upregulates VEGF and OPN expressions in HCC cells [16, 17], but also strengthens their stemness characteristics [10]. Other literatures also support that increased matrix stiffness elevates the expression of integrin β1, and is positively correlated with the invasion and metastasis of HCC patients with cirrhosis [12]. Additionally, higher matrix stiffness can alter chemotherapeutic responses of HCC cells [1]. The formation of tumor pre-metastatic niche, which occurs in the distant target organ/tissue, is a critical molecule event at the late stage of tumor metastasis, and determines the implementation of distant metastasis. Generally, pre-metastatic niche resembles as the fertile “soil” and assists circulating tumor cells settlement in target organ/tissue and facilitates tumor distant metastasis [18]. In these years, the identified molecules and cells in distant metastasis tissue of different tumor animal models including the primary tumor-derived soluble factors, vesicles, exosome and bone marrow derived cells (BMDCs), etc. gradually confirmed the existence of pre-metastasis niche in the most types of malignant tumors [18, 19]. However, little is known about the linkage between matrix stiffness and pre-metastatic niche in HCC. Lysyl oxidase (LOX) family is composed of LOX, LOXL1, LOXL2, LOXL3 and LOXL4. All of these five members have highly conserved C-terminal domain that contains copper binding motif, lysine tryosylquinone residues and a cytokine receptor-like domain, therefore they exhibit similar catalytic activity [20]. However, their amino-terminal regions are different, determine their different roles in protein-protein interaction [21] To date, only few soluble factors such as tumor secreted LOXL2 [22], exosomes [23] exhibit important pathological roles in formation of pre-metastatic niche in HCC metastasis. FoxM1b stimulates the expressions of LOX and LOXL2 to induce pre-metastatic niche formation in the lung of HCC animal model [24]. Although higher matrix stiffness forces the expression of LOX in HCC as described previously [16], it remains largely unknown that which member of LOX family in HCC plays dominating function in matrix stiffness-induced effects on pre-metastatic niche. In the study, we investigated the expression of LOX family members in HCC cells grown on different substrate stiffness and its regulation mechanism, and subsequently analyzed possible biological effects of LOXL2 on pre-metastatic niche formation such as BMDCs recruitment, fibronectin production, MMPs and CXCL12 expression,cell adhesion, etc.

## Methods

### A gel-based culture system with tunable substrate stiffness

A gel-based culture system with tunable substrate stiffness was established as our previous study [16].

### Cells and cell culture

MHCC97H, a highly metastatic type of HCC cell, was established at the Liver Cancer Institute of Fudan University. A lowly metastatic HCC cell Hep3B and human embryo lung fibroblast (HELF) were obtained from the Cell Bank of Shanghai Institute of Biochemistry and Cell Biology, CAS. Approximately 1 × 10^6^ HCC cells in 0.3 ml of medium were spread evenly onto COL1-coated gel substrate with tunable stiffness in dish and cultured for 2 h at room temperature. Subsequently, 12 ml medium was added into dish and the cells were cultured in incubator for 48 h. MHCC97H cells and HELF cells were all grown in Dulbecco’s Modified Eagle’s Medium (DMEM, Gibco) supplemented with 10% fetal bovine serum (FBS; Biowest) and 1% penicillin/streptomycin (Gibco), and Hep3B cells in minimum essential medium (MEM, Gibco) with 10% fetal FBS and 1% penicillin/streptomycin.

### Collection of conditioned medium (CM) from HCC cells

HCC cells transfected with LV-scramble (NC) or LV-LOXL2-OE were cultured in serum-free DMEM for 24 h, and their culture supernatants were collected and then sterilized by a 0.2 μm filter (Millipore, Schwalbach, Germany). The collected CM were stored at − 80 °C for further use.

### Recombinant plasmid construction of c-JUN and transient transfection

The shRNA sequence (CcggcgGACCTTATGGCTACAGTAAcg CTGTAGCCATAAGGTCCGTTTTTg) targeting human c-JUN gene was designed by the Shanghai GeneChem, Co. Ltd., China. The uppercase letters and lowercase letters represented c-JUN-specific sequence and hairpin sequences, respectively. The double-strand DNA was cloned into GV248 vector and recombinant plasmid expressing c-JUN-shRNA was constructed. HCC cells were transfected with recombinant plasmid shRNA-c-JUN using lipofectamine 2000, and the transfected HCC cells were collected after 48 h culture.

### Western blot

Total proteins of the collected cells were extracted in lysis buffer containing RIPA buffer (Beyotime, China), 1 mM phenylmethanesulfonyl fluoride (Beyotime, China), and 10% PhosSTOP (Roche, Switzerland). Approximately 20 μg total protein was loaded and separated by 12% SDS-PAGE, then transferred onto a polyvinylidene difluoride membrane (Millipore, USA). Subsequently, the PVDF membrane was blocked in TBS/Tween with 5% fat-free milk and incubated with the following primary antibodies: GAPDH (1:1000, Cell Signal Technology), LOX (1:1000, Abcam), LOXL1 (1:1000, Abcam), LOXL2 (1:1000, Abcam; 1:2000, Novus Biologicals), LOXL3 (1:100, Santa Cruz), LOXL4 (1:500, Abnova), c-JUN (1:1000, Proteintech), p-c-JUN (Ser73) (1:1000, Cell Signal Technology), JNK, p-JNK (Thr183/Tyr185) (1:1000, Cell Signal Technology), MMP2 (1:1000, Abcam), MMP9 (1:1000, Cell Signal Technology). The membrane was further incubated with HRP-conjugated secondary antibody (1:4000, Proteintech). Finally, the target protein band was visualized using an electrochemiluminescence kit (Thermo).

### Quantitative reverse transcription polymerase chain reaction (qRT-PCR)

Total RNA was extracted from HCC cells using Trizol reagent (Invitrogen). mRNA was reverse-transcribed into cDNA using a RevertAid/First Strand Synthesis Kit (Thermo Scientific), and then cDNA template was further amplified with gene specific primer and SYBR Green PCR Master Mix kit (Invitrogen). Three replicates were set for each primer in each group, the relative mRNA expression was normalized to GAPDH and reported as 2^-△△Ct^. The primer sequences of the detected genes are listed in Table 1.

**Table 1:** Primer sequence used for qRT-PCR

Gene symbol	Forward primer (5′-3′)	Reverse primer (5′-3′)
LOX	TTACCCAGCCGACCAAGATA	CCTTCAGCCACTCTCCTCTG
LOXL1	TACGATGTGCGGGTGCTAC	ATGCTGTGGTAATGCTGGTG
LOXL2	AGGATGTCGGTGTGGTGTG	TTGCGGTAGGTTGAGAGGAT
LOXL3	TGGAGTTCTATCGTGCCAATGA	CCTGAGGCTTCGACTGTTGT
LOXL4	ACTGTAGGCTGCTGGGACAC	GGTTCACAATCACCTGGAAGA
MMP2	GTTCATTTGGCGGACTGT	AGGGTGCTGGCTGAGTAG
MMP9	GGGACGCAGACATCGTCATC	TCGTCATCGTCGAAATGGGC
CXCL12	GTTCAAAGCCAGCGTC	TAGTTCACCCCAAAGGA
GAPDH	CTCCTCCACCTTTGACGC	CCACCACCCTGTTGCTGT

### Stable knockdown expression of integrin β1, integrin α5 and overexpression of LOXL2 in HCC cells using lentiviral-mediated RNAi/overexpression technology

The interference sequences targeting human integrin β1 (ITGβ1, 5’-CCTCCAGATGACATAGAAA-3′), integrin α5 (ITGA5, 5’-TCAGGAACGAGTCAGAATT-3′), and human LOXL2 overexpression sequence (see Additional file 1) were designed by the Shanghai GeneChem, Co. Ltd., China. Target sequence to integrin β1 or α5 genes was cloned into the plasmid pGCSIL, and LOXL2 overexpression sequence was cloned into GV416 vector. LV-integrin-shRNA and LV-LOXL2-OE were obtained by co-transfecting above plasmids and packing plasmids Helper1.0 and Helper2.0 into 293 T cells. The viral supernatant (at multiplicity of infection = 10) containing Eni.S and 5 μg/ml polybrene was added into HCC cells. After 12 h, the transfection solution was replaced with normal culture medium for continuing culture to obtain stably-transfected HCC cells. Assessment of transfection efficiency see Additional file 2: Figure S1.

### Immunohistochemistry analysis

The detailed procedure of immunohistochemistry was described in our previous work [16]. Cell slides were incubated with primary antibody against LOXL2 (1:400, Abcam), c-JUN (1:50, Proteintech) at 4 °C overnight, and then reacted with secondary antibody conjugated with HRP (1:200, DingguoBio, Beijing). Staining intensity and the percentage of the stained tissues were scored by two independent observers. Photographs of four representative sites were captured under high-power magnification (× 200) by the Leica QWin Plus v3 software with identical setting parameters. The density of positive staining was measured by Image-Pro Plus v6.2 software (Media Cybernetics Inc., USA). Integrated optical density of the positive stains in each photograph was measured, and its ratio to the total area of each photograph was calculated.

### JNK inhibition assay

JNK-IN-8 (MedChem Express, USA) is a selective JNK1/2/3 inhibitor, and inhibits phosphorylation of downstream molecule c-JUN. JNK-IN-8 dissolved in DMSO was diluted into 1 μM work solution with complete culture medium, and the same amount of DMSO was set as the control (V_DMSO_:V _(complete culture medium)_ = 0.5‰). HCC cells grown on high stiffness substrates were treated with JNK-IN-8 for 48 h.

### Cell motility assay

Approximately 4 × 10^5^ HCC cells suspended in 200 μl serum-free DMEM were placed into the upper chamber of a Bodyen Chamber (Millipore), 250 μl DMEM containing 20% FBS and 250 μl CM-LV-LOXL2-OE were mixed and placed in lower chamber. After 48 h incubation, the migrating cells in the membrane were fixed with 4% paraformaldehyde for 30 min, and then stained in crystal violet for 30 min. The number of cells in six random fields were counted under a light microscope.

### BMCs isolation and BMCs recruitment assay in vitro

The cells in femurs and tibias of mice were harvested by PBS flushing, and then treated with Red Blood Cell Lysis Buffer (Beyotime Biotechnology) to isolate bone marrow cells (BMCs). For BMCs Invasion assay, Transwell inserts (Millipore) were coated with Matrigel (BD Biosciences) at 37 °C for 2 h. 1 × 10^6^ fresh BMCs suspended in 200 μl serum-free DMEM were seeded into the upper chamber. 500 μl of mixed medium as a chemoattractant (250 μl DMEM with 10% FBS and 250 μl CM-LV-LOXL2-OE) were placed in lower chamber. After 24 h incubation, cells that invaded through the membrane were founded in the lower chamber suspension and counted. The cell motility assay was performed in a similar method, except that the cells were seeded into the uncoated inserts.

### Cell adhesion assay

After HELF cells grew and reached 70% confluence in 6 well plate, they were treated with CM-LV-LOXL2-OE. When the treated HELF cells were close to 100% confluence, 2 × 10^5^ GFP-labeled HCC cells were put upon the treated HELF cells. Twenty-four hours later, the attached MHCC97H cells on the surface of HELF cells were photographed using a fluorescence microscopy (Leica). The number of attached HCC cells was measured by counting six random microscope fields (× 200), and data were expressed as a means ± SEM.

### Statistical analysis

Data analysis was performed using SPSS 20.0 statistical software (SPSS Inc., Chicago, IL). All data were expressed as mean ± standard error of the mean (SEM). Statistical analysis was performed by Student’s t-test. *p* < 0.05 indicating statistical significance. All experiments have been performed in triplicate.

## Results

### The expression levels of LOX family members in HCC cells grown on different stiffness substrates

**Fig. 1:**
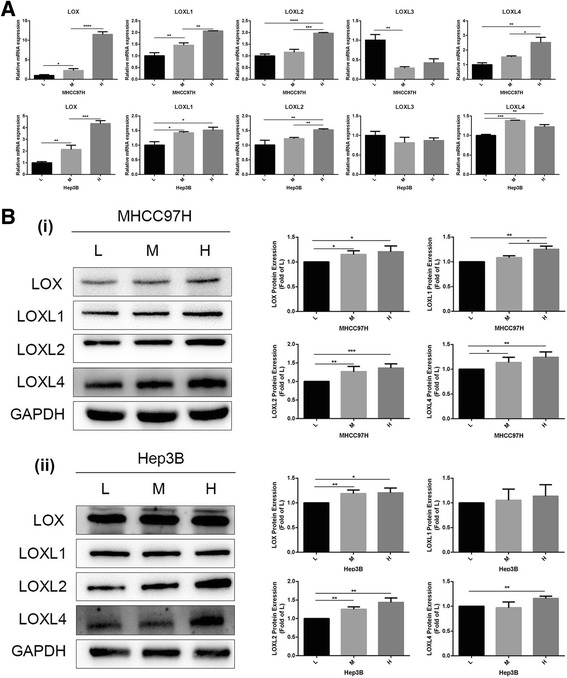
Expressions of the LOX family members in HCC cells under diverse stiffness stimulation. (**a**) mRNA expression levels of LOX, LOXL1, LOXL2, LOXL3 and LOXL4 in MHCC97H and Hep3B cells grown on 6 KPa (L), 10 KPa (M), and 16 KPa (H) stiffness substrate. (**b**) (i, ii) The protein expression level of LOX, LOXL1, LOXL2, LOXL4 in MHCC97H and Hep3B cells grown on 6 KPa (L), 10 KPa (M), and 16 KPa (H) stiffness substrate. LOXL3 was not detected at the protein level. Error bar represents SEM, *p < 0.05, **p < 0.01 and ***p < 0.001

**Fig. 2:**
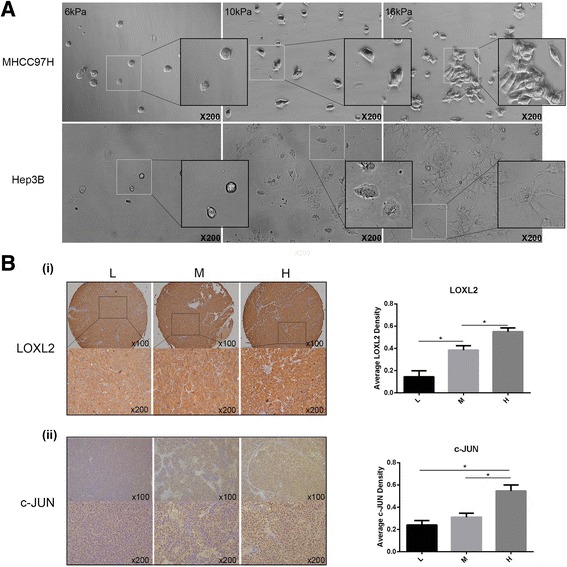
Morphological changes of HCC cells under different stiffness stimulation and the expression of LOXL2 and c-JUN in HCC tissue with different liver stiffness backgrounds. (**a**) Morphology of MHCC97H and Hep3B cells cultured on 6 kPa, 10 kPa, and 16 kPa stiffness substrates. (**b**) (i) The expression levels of LOXL2 in HCC tissues with normal liver stiffness backgrounds (groups L), medium liver stiffness backgrounds (group M) and high liver stiffness backgrounds (group H). (ii) The expression levels of c-JUN in HCC tissues with different liver stiffness backgrounds. Error bar represents SEM, *p < 0.05

We used 6 KPa, 10 KPa, and 16 KPa, as our previous report [16], to represent the stiffness values of normal liver tissue, fibrosis, and cirrhosis tissue. We first detected mRNA and protein expressions of LOX family members in HCC cells growing on different stiffness substrates, and found that the expressions of LOX, LOXL1, LOXL2 and LOXL4 except for LOXL3 were all significantly upregulated in MHCC97H and Hep3B cells under higher stiffness stimulation (Fig. 1a, b). LOXL3 expression was not detected at the protein level. According to the expression levels of LOX family members in HCC cells under different stiffness stimulation and the significance role of LOX family members in pre-metastatic niche [22, 25], we speculated that the secreted LOXL2 from HCC cells might mediate the linkage between higher matrix stiffness and pre-metastatic niche, and facilitate tumor distant metastasis. In addition, higher matrix stiffness stimulation significantly altered shape and spread area of HCC cells. MHCC97H and Hep3B cells grown on 10, 16 KPa stiffness substrate exhibited an extension and expansion morphology, different from small round shape on 6 KPa stiffness substrate (Fig. 2a). Morphological alteration perhaps means a great change in their malignant characteristics. To confirm LOXL2 expression level in vivo, we further applied a HCC tissue microarray established previously [16] to analyze the effect of liver matrix stiffness on LOXL2 expression. Our data showed that LOXL2 highly expressed in HCC tissues with high and medium liver stiffness backgrounds as compared with that of HCC tissue with normal liver stiffness background (Fig. 2b (i)), in agreement with the results in vitro.

### Integrin β1/α5/JNK/c-JUN signaling pathway may be involved in matrix stiffness-induced LOXL2 upregulation in HCC cells

**Fig. 3:**
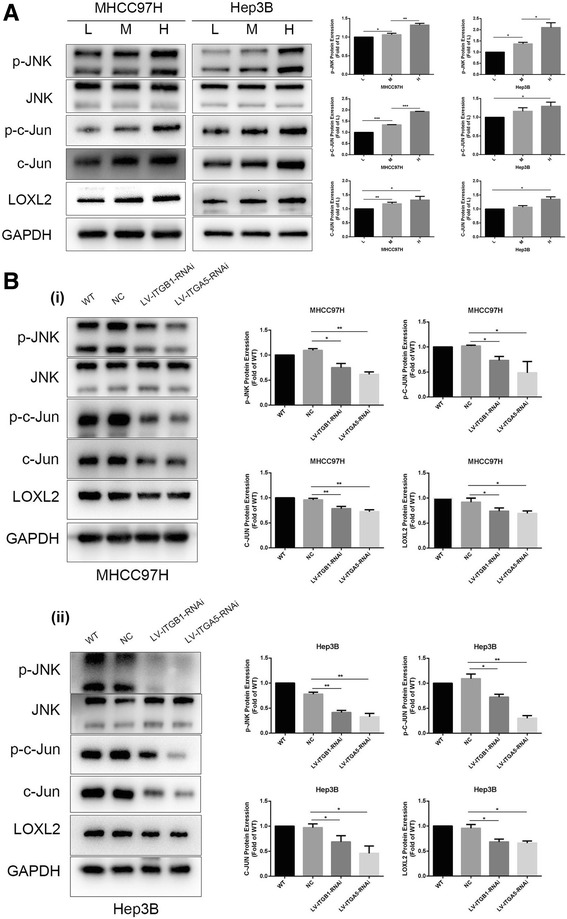
Higher matrix stiffness upregulated LOXL2 expression in HCC cells via activating integrin β1/α5/JNK/c-JUN signaling pathway. (**a**) Increased matrix stiffness upregulated LOXL2 expression and activated JNK/c-JUN signaling pathway in MHCC97H and Hep3B cells. (**b**) (i, ii) Knockdown of integrin β1 or integrin α5 obviously suppressed the expression levels of p-JNK, p-c-JUN, c-JUN and LOXL2 in HCC cells grown on 16 KPa stiffness substrate. Error bar represents SEM, *p < 0.05, **p < 0.01 and ***p < 0.001

Higher matrix stiffness upregulated the expression of LOXL2 in MHCC97H and Hep3B cells, simultaneously significantly improved the phosphorylation levels of JNK, the phosphorylation and expression levels of c-JUN (Fig. 3a), illustrating that high matrix stiffness might activate the JNK/c-JUN signaling pathway. We further analyzed the expression of signal molecule of c-JUN in HCC tissues, and found that the expression level of c-JUN elevated as liver stiffness background increased. (Fig. 2b (ii)). Given that JNK/c-JUN pathway is associated with LOX expression [26, 27], we conjectured that activation of JNK/c-JUN pathway might participate in high matrix stiffness-induced LOXL2 upregulation. Previously, we reported that integrin β1 and α5 as major bridge molecules delivered stiffness signal into HCC cells [16]. Here, knockdown of integrin β1 or integrin α5 suppressed the phosphorylation levels of JNK and c-JUN obviously in HCC cells grown on higher stiffness substrate, also downregulated the expression of LOXL2 and c-JUN (Fig. 3b), demonstrating that higher matrix stiffness-activated JNK/c-JUN pathway was partially reversed. Thereby, activation of integrin β1/α5-JNK/c-JUN pathway is required for higher matrix stiffness-induced LOXL2 upregulation in HCC cells.

### JNK inhibitor and shRNA-c-JUN remarkably suppress LOXL2 expression and JNK/c-JUN pathway activation in HCC cells grown on high stiffness substrate

**Fig. 4:**
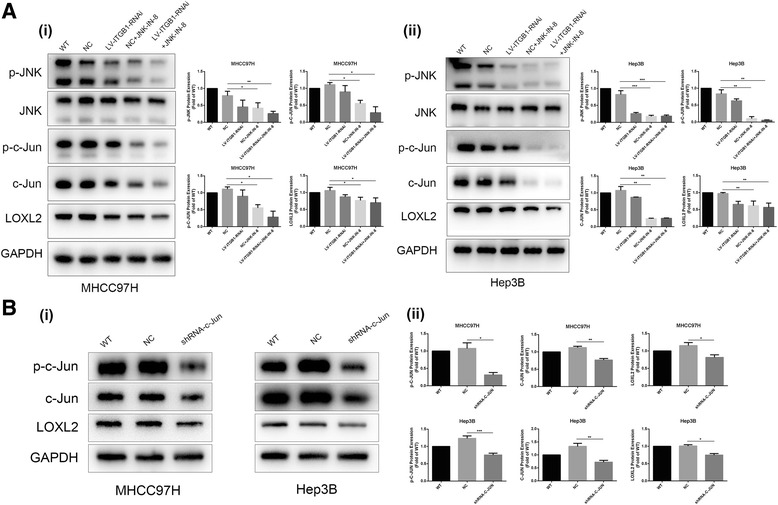
JNK inhibitor and shRNA-c-JUN remarkably attenuated LOXL2 expression and JNK/c-JUN pathway activation in HCC cells grown on higher stiffness substrate. (**a**) A co-inhibitor of JNK and integrin β obviously decreased phosphorylation levels of JNK and c-JUN and expression of LOXL2 as compared with JNK inhibitor alone or integrin β1 alone. (**b**) Knockdown of c-JUN inhibited LOXL2 expression significantly in MHCC97H and Hep3B cells grown 16 KPa stiffness substrate. Error bar represents SEM, *p < 0.05, **p < 0.01 and ***p < 0.001

JNK-IN-8, a selective JNK inhibitor, inhibits phosphorylation of its downstream molecule c-JUN [28]. We used the JNK inhibitor to treat HCC cells under higher stiffness stimulation and further measured LOXL2 expression and the activation of JNK/c-JUN pathway. As shown in Fig. 4a, JNK inhibitor sharply attenuated the expression of p-JNK, p-c-JUN, c-JUN as well as LOXL2 in HCC cells grown on higher stiffness substrate, and shRNA-c-JUN also decreased LOXL2 expression remarkably (Fig. 4b). These above results strongly support that JNK/c-JUN pathway specifically modulates matrix stiffness-induced LOXL2 upregulation. Simultaneously, a co-inhibitor of JNK and integrin β showed a more pronounced decrease in phosphorylation levels of JNK and c-JUN and expression of LOXL2 as compared with JNK inhibitor or integrin β1 alone (Fig. 4b), further confirming that integrin β1 and JNK/c-JUN pathway contributed to matrix stiffness-induced LOXL2 upregulation. Taken together, we concluded that high matrix stiffness indeed activated the JNK/c-JUN pathway in HCC cells and then upregulated LOXL2 expression.

### LOXL2 increases motility and invasion of BMCs, simultaneously improves MMP9 and CXCL12 expression, as well as fibronectin production in lung fibroblasts, which assists HCC cells settlement on them

**Fig. 5:**
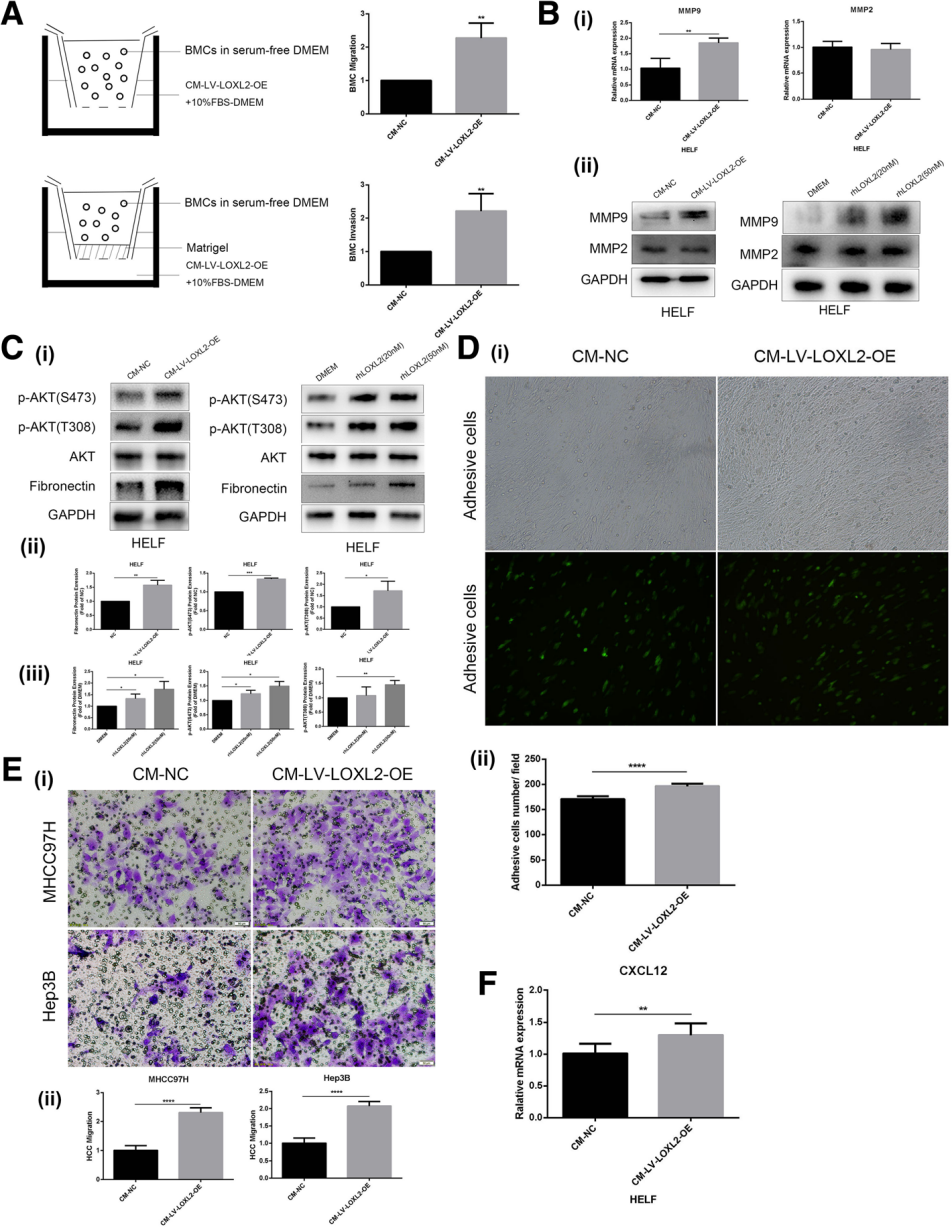
LOXL2 increases motility and invasion of BMCs, simultaneously improves MMP9 and CXCL12 expression, as well as fibronectin production in lung fibroblasts, which assists assists the formation of pre-metastatic niche. (**a**) CM-LV-LOXL2-OE as a chemoattractant significantly increased BMCs migration and invasion compared to the control. (**b**) (i, ii) CM-LV-LOXL2-OE or rhLOXL2 all upregulated MMP9 expression in HELF cells, but had no effect on MMP2 expression. (**c**) (i-iii) CM-LV-LOXL2-OE or rhLOXL2C activated AKT signaling pathway in HELF cells and upregulated fibronectin expression. (**d**) (i,ii) The number of HCC cells adhered to surface of lung fibroblasts treated with CM-LV-LOXL2-OE was significantly higher than that of the control cells. (**e**) (i,ii) CM-LV-LOXL2-OE as chemoattractant increased the number of migrated HCC cells. (**f**) CM-LV-LOXL2-OE improved CXCL12 expression in lung fibroblasts. Error bar represents SEM, *p < 0.05, **p < 0.01, ***p < 0.001 and ****p < 0.0001

BMDCs recruitment and matrix remodeling are two important characteristics of pre-metastatic niche formation. We assessed the effects of LOXL2 on BMDCs recruitment and matrix remodeling in vitro by BMCs migration/invasion assay and MMP expression. As shown in Fig. 5a, the number of migrated BMCs or invaded BMCs in CM-LV-LOXL2-OE group was significantly higher than that of CM-NC group. LOXL2 in CM as chemoattractant increased motility and invasion of BMCs, implicating a significant role of LOXL2 in BMCs recruitment. We analyzed MMP9 and MMP2 expression in lung fibroblasts treated with CM-LV-LOXL2-OE and found that CM-LV-LOXL2-OE significantly elevated MMP9 expression, but had no effect on MMP2 expression (Fig. 5b). A recombinant protein LOXL2 (rhLOXL2) also showed same effects on MMP9 and MMP2 expressions, suggesting that the secreted LOXL2 upregulates MMP9 expression in lung fibroblasts and is in favor of matrix remodeling in lung tissue. On the other hand, CM-LV-LOXL2-OE remarkably upregulated fibronectin expression in lung fibroblasts, simultaneously it increased phosphorylation levels of AKT (Ser473 and Thr308) significantly (Fig. 5c (i, ii)). Furthermore, rhLOXL2 with the concentration of 20 nM and 50 nM obviously elevated phosphorylation levels of AKT and fibronectin expression in lung fibroblasts (Fig. 5c (i, iii)), demonstrating that AKT pathway may participate in LOXL2-induced fibronectin upregulation. Undoubtedly, both MMP9 upregulation and matrix protein deposition created a suitable “soil” in lung for circulating tumor cells colonization and growth. Fibronectin is a glycoprotein of the extracellular matrix that can bind to integrins [29], and plays a crucial role in cell adhesion, growth, migration and differentiation. Our data showed that the number of HCC cells adhered to surface of lung fibroblasts treated with CM-LV-LOXL2-OE was significantly higher than that of the control cells (Fig. 5d), defining the roles of LOXL2 in promoting HCC cells settlement on pre-metastatic niche. Except the above results, we also found that CM-LV-LOXL2-OE as chemoattractant increased the number of migrated HCC cells (Fig. 5e), and improve CXCL12 expression in lung fibroblasts (Fig. 5f), revealing that the secreted LOXL2 also facilitated the motility of HCC cells and strengthened CTCs settlement on the remodeled matrix “soil”. Accordingly, the secreted LOXL2 form HCC facilitates the formation of pre-metastatic niche and the accomplishment of tumor distant metastasis.

## Discussion

Although LOX family members increase extracellular matrix stiffness and accelerate tumor progression [22, 30, 31], the effects of matrix stiffness on LOX family member expression are not yet understood. LOX catalyzes the cross-linking of collagen and elastin for maintaining the rigidity and structural stability of the extracellular matrix (ECM) [32, 33]. Overexpressed LOX in cancers is related to tumorigenesis, tumor progression and metastasis [21, 34–37]. LOX and LOXL4 participate in tumor microenvironment remodeling and the formation of pre-metastatic niches [38, 39]. LOXL2 has similar biological activities in ECM remodeling as LOX, and its overexpression indicates poor prognosis in patients with colon and esophageal squamous cell carcinoma [40], as well as gastric cancer and breast cancer [41, 42]. Highly invasive breast cancer cells instead of non-metastatic cells present obviously high expression of LOXL2 [43, 44]. LOXL2 may be a promising therapeutic target for preventing invasive/metastatic of breast cancer [41]. LOX and LOXL2 as primary tumor-derived soluble factors contribute to formation of pre-metastatic niches in distant target organs [22, 35, 39, 45]. However, little is known about the significance of matrix stiffness-upregulated LOXL2 in pre-metastatic niches formation of HCC.

Higher liver stiffness, as a common physical attribute of chronical liver disease, always accompanies with HCC development, and it is also involved in the regulation of HCC invasion and metastasis [12, 46]. Wong et al. [22] proposes that hypoxic or TGF-β-induced LOXL2 stiffens tumor tissue, and then facilitates the formation of pre-metastasis niche in HCC. Hereby, we speculated that LOXL2 might mediate matrix stiffness-induced effects on pre-metastatic niche. Unlike previous reports on biochemical signal stimuli, the initiator of stimulation in the study was physical mechanical signal. Higher matrix stiffness stimulation remarkably upregulated the expression of LOX family members except LOXL3 in HCC cells. Based on the expression levels of matrix stiffness-induced LOX members and their potential significance in pre-metastatic niche, we determined to explore underlying molecular mechanism of higher matrix stiffness-induced LOXL2 upregulation, and analyze its significance in pre-metastatic niche formation of HCC. Several literatures have reported that JNK/c-JUN pathway takes part in the regulation of LOXL2 expression [26, 27]. Our results demonstrated that high matrix stiffness activated the integrin/JNK/c-JUN signaling pathway and increased LOXL2 expression in HCC cells. Except that, JNK inhibitor partially suppressed phosphorylation levels of JNK and c-JUN, and the expression of LOXL2 in HCC cells grown on higher stiffness substrate. ShRNA-c-JUN also downregulated LOXL2 expression significantly. Accordingly, there may be two regulating pathways for higher matrix stiffness-upregulated LOXL2 expression. One way is that increased matrix stiffness activates the phosphorylation JNK and c-JUN via integrinβ1/α5, and result in high expression of LOXL2. The other is that higher matrix stiffness directly influences the expression of c-JUN through integrinβ1/α5 and then regulate LOXL2. Although integrin/JNK/c-JUN pathway contributes to higher matrix stiffness-upregulated LOXL2 in HCC cells, we are still unable to exclude the activation of other signal pathways in this regulating process. Inhibition of integrin β1 and α5 partially attenuated phosphorylation level of JNK and c-JUN in HCC cell under higher stiffness stimulation (Fig. 3), suggesting that other pathways may participate in matrix stiffness-upregulated LOXL2 in HCC cells. Additionally, the results in the study also implied indirectly that β1 integrin family-dependent signaling may determine the functional role of JNK in cancer cells [47].

BMDCs recruitment and matrix remodeling are two common pathological changes in metastatic target organ during the formation of tumor pre-metastatic niche. TDSFs float to distant tissues with blood circulation and promote BMDCs recruitment. The recruited BMDCs interact with resident stromal cells in target organ and produce various growth factors, integrins, chemokines, inflammatory mediators, pro-angiogenic molecules, remodeling matrix microenvironment in target tissue, to facilitate tumor cells colonization and proliferation [48, 49]. In the study, LOXL2 in CM as chemoattractant increased the number of migrated BMCs or invaded BMCs, indicating a significant role of LOXL2 in BMCs recruitment. Additionally, CM-LV-LOXL2-OE and rhLOXL2 also elevated MMP9 expression and fibronectin production significantly in lung fibroblasts, and activation of Akt pathway might participate in LOXL2-induced fibronectin upregulation. Both of MMP9 upregulation and matrix protein deposition triggered matrix remodeling and created a suitable “soil” in lung tissue for circulating tumor cell colonization and growth. Fibronectin is a high-molecular weight glycoprotein of ECM that binds to transmembrane receptor protein integrin, and fibronectin-integrin signaling is closely related to ECM remodeling [50]. Fibronectin implicates on cell adhesion, growth, migration and differentiation [29], as well as carcinoma development [51]. Additionally, fibronectin exerts a crucial role in pre-metastatic niche formation by generating a suitable extracellular matrix “soil” and promoting CTCs settlement on them. TDSFs released by primary tumor promote HPCs recruitment at distant target organ, and recruited HPCs localize to areas of increased fibronectin which is newly synthesized by resident fibroblasts in distant organ. The HPCs, along with fibronectin and associated stromal cells, alter the local microenvironment, leading to the activation of other integrins and the secretion of chemokines such as CXCL12 that promote attachment, survival and growth of tumor cells [52, 53]. Our data revealed that the number of HCC cells adhered to surface of the treated lung fibroblasts with CM-LV-LOXL2-OE was obviously increased compared with that of the control cells, defining the roles of LOXL2 in promoting HCC cells settlement on pre-metastatic niche. Besides, CM-LV-LOXL2-OE as chemoattractant increased the number of migrated HCC cells, and improve the expression of chemokine CXCL12 in lung fibroblasts, suggesting that the secreted LOXL2 also facilitated the motility ability of HCC cells and strengthened CTCs settlement on the remodeled matrix “soil”. Although the data in vitro support a significant role of LOXL2 in facilitating formation of pre-metastatic niche, it is still necessary to further confirm its roles in animal model in the following study.

## Conclusion

**Fig. 6:**
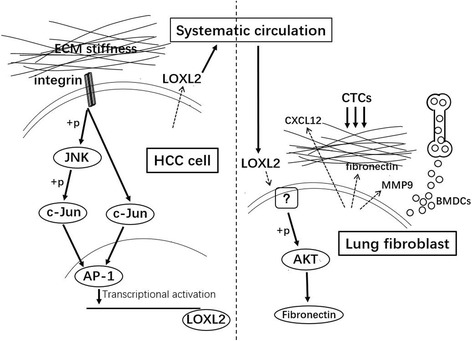
The proposed mechanism by which higher matrix stiffness upregulates LOXL2 expression in HCC cells, and the secreted LOXL2 promotes fibronectin production, MMP9 and CXCL12 expression and BMDCs recruitment to assist pre-metastatic niche formation

Integrin β1/α5/JNK/c-JUN signaling pathway participates in higher matrix stiffness-induced LOXL2 upregulation in HCC cells. The secreted LOXL2 promotes fibronectin production, MMP9 and CXCL12 expression and BMDCs recruitment to assist pre-metastatic niche formation (Fig. 6).

## Additional files

Additional file 1: Sequence of human LOXL2 overexpression. (DOCX 16 kb)

Additional file 2: **Figure S1.** Assessment of transfection efficiency. (A) (i) The transfected HCC cells with LV-LOXL2-OE. (ii) The expression level of LOXL2 in MHCC97H cells transfected with LV-LOXL2-OE. (B) The expression level of integrin β1 in HCC cells transfected with LV-ITGB1-RNAi. (C) The expression level of integrin α5 in HCC cells transfected with LV-ITGA5-RNAi. (JPG 336 kb)

## Abbreviations

AP-1: Activator protein 1
CM: Conditioned medium
COL1: Collagen 1
CTC: Circulating tumor cell
CXCL12: C-X-C motif chemokine ligand 12
ECM: Extracellular matrixi
GAPDH: Glyceraldehyde-3-phosphate dehydrogenase
HCC: Hepatocellular carcinoma
HELF: Human embryo lung fibroblast
HPC: Haematopoietic progenitor cell
JNK: c-Jun N-terminal kinase
LOX: Lysyl oxidase
LOXL1: Lysyl oxidase like 1
LOXL2: Lysyl oxidase like 2
LOXL3: Lysyl oxidase like 3
LOXL4: Lysyl oxidase like 4
MMP2: Matrix metalloproteinnase 2
MMP9: Matrix metalloproteinase 9
NC: Negative control
OPN: Osteopontin
qRT-PCR: Quantitative reverse transcription polymerase chain reaction
rhLOXL2: Recombinant human Lysyl oxidase like 2
TDSF: Tumor derived soluble factor
TGF-β: Transforming growth factor beta
VEGF: Vascular endothelial growth factor
