# Supplementary material for: Matrix stiffness-upregulated LOXL2 promotes fibronectin production, MMP9 and CXCL12 expression and BMDCs recruitment to assist pre-metastatic niche formation
Source: J Exp Clin Cancer Res. 2018 May 4;37:99. doi: 10.1186/s13046-018-0761-z (PMC5935912; doi:10.1186/s13046-018-0761-z)
Supplement: Supplementary file 1 — Sequence of human LOXL2 overexpression. (DOCX 16 kb) [file 13046_2018_761_MOESM1_ESM.docx]

**Additional file 1**

**materials and Methods**

Human LOXL2 overexpression sequence:

GCAAGGGAGAAGGGCCCATCTGGTTAGACAATCTCCACTGTACTGGCAACGAGGCGACCCTTGCAGCATGCACCTCCAATGGCTGGGGCGTCACTGACTGCAAGCACACGGAGGATGTCGGTGTGGTGTGCAGCGACAAAAGGATTCCTGGGTTCAAATTTGACAATTCGTTGATCAACCAGATAGAGAACCTGAATATCCAGGTGGAGGACATTCGGATTCGAGCCATCCTCTCAACCTACCGCAAGCGCACCCCAGTGATGGAGGGCTACGTGGAGGTGAAGGAGGGCAAGACCTGGAAGCAGATCTGTGACAAGCACTGGACGGCCAAGAATTCCCGCGTGGTCTGCGGCATGTTTGGCTTCCCTGGGGAGAGGACATACAATACCAAAGTGTACAAAATGTTTGCCTCACGGAGGAAGCAGCGCTACTGGCCATTCTCCATGGACTGCACCGGCACAGAGGCCCACATCTCCAGCTGCAAGCTGGGCCCCCAGGTGTCACTGGACCCCATGAAGAATGTCACCTGCGAGAATGGGCTACCGGCCGTGGTGAGTTGTGTGCCTGGGCAGGTCTTCAGCCCTGACGGACCCTCGAGATTCCGGAAAGCGTACAAGCCAGAGCAACCCCTGGTGCGACTGAGAGGCGGTGCCTACATCGGGGAGGGCCGCGTGGAGGTGCTCAAAAATGGAGAGTGGGGGACCGTCTGCGACGACAAGTGGGACCTGGTGTCGGCCAGTGTGGTCTGCAGAGAGCTGGGCTTTGGGAGTGCCAAAGAGGCAGTCACTGGCTCCCGACTGGGGCAAGGGATCGGACCCATCCACCTCAACGAGATCCAGTGCACAGGCAATGAGAAGTCCATTATAGACTGCAAGTTCAATGCCGAGTCTCAGGGCTGCAACCACGAGGAGGATGCTGGTGTGAGATGCAACACCCCTGCCATGGGCTTGCAGAAGAAGCTGCGCCTGAACGGCGGCCGCAATCCCTACGAGGGCCGAGTGGAGGTGCTGGTGGAGAGAAACGGGTCCCTTGTGTGGGGGATGGTGTGTGGCCAAAACTGGGGCATCGTGGAGGCCATGGTGGTCTGCCGCCAGCTGGGCCTGGGATTCGCCAGCAACGCCTTCCAGGAGACCTGGTATTGGCACGGAGATGTCAACAGCAACAAAGTGGTCATGAGTGGAGTGAAGTGCTCGGGAACGGAGCTGTCCCTGGCGCACTGCCGCCACGACGGGGAGGACGTGGCCTGCCCCCAGGGCGGAGTGCAGTACGGGGCCGGAGTTGCCTGCTCAGAAACCGCCCCTGACCTGGTCCTCAATGCGGAGATGGTGCAGCAGACCACCTACCTGGAGGACCGGCCCATGTTCATGCTGCAGTGTGCCATGGAGGAGAACTGCCTCTCGGCCTCAGCCGCGCAGACCGACCCCACCACGGGCTACCGCCGGCTCCTGCGCTTCTCCTCCCAGATCCACAACAATGGCCAGTCCGACTTCCGGCCCAAGAACGGCCGCCACGCGTGGATCTGGCACGACTGTCACAGGCACTACCACAGCATGGAGGTGTTCACCCACTATGACCTGCTGAACCTCAATGGCACCAAGGTGGCAGAGGGCCACAAGGCCAGCTTCTGCTTGGAGGACACAGAATGTGAAGGAGACATCCAGAAGAATTACGAGTGTGCCAACTTCGGCGATCAGGGCATCACCATGGGCTGCTGGGACATGTACCGCCATGACATCGACTGCCAGTGGGTTGACATCACTGACGTGCCCCCTGGAGACTACCTGTTCCAGGTTGTTATTAACCCCAACTTCGAGGTTGCAGAATCCGATTACTCCAACAACATCATGAAATGCAGGAGCCGCTATGACGGCCACCGCATCTGGATGTACAACTGCCACATAGGTGGTTCCTTCAGCGAAGAGACGGAAAAAAAGTTTGAGCACTTCAGCGGGCTCTTAAACAACCAGCTGTCCCCGCAGGGATCCCCGGGTAGCGCTATGGACTACAAGGATGACGATGACAAGGATTACAAAGACGACGATGATAAGGACTATAAGGATGATGACGACAAATGAGCTAGCTGTTCTTTCCTGCGTTATCCCCTGATTCTGTGGATAACCGTATTACCGCCATGCATTAGTTATTAATAGTAATCAATTACGGGGTCATTAGTTCATAGCCCATATATGGAGTTCCGCGTTACATAACTTACGGTAAATGGCCCGCCTGGCTGACCGCCCAACGACCCCCGCCCATTGACGTCAATAATGACGTATGTTCCCATA.

Red highlighted part is the inserted sequence, and the black underlined part is the sticky end.
