# Supplementary figures and images for: Matrix stiffness-upregulated LOXL2 promotes fibronectin production, MMP9 and CXCL12 expression and BMDCs recruitment to assist pre-metastatic niche formation
Source: J Exp Clin Cancer Res. 2018 May 4;37:99. doi: 10.1186/s13046-018-0761-z (PMC5935912; doi:10.1186/s13046-018-0761-z)

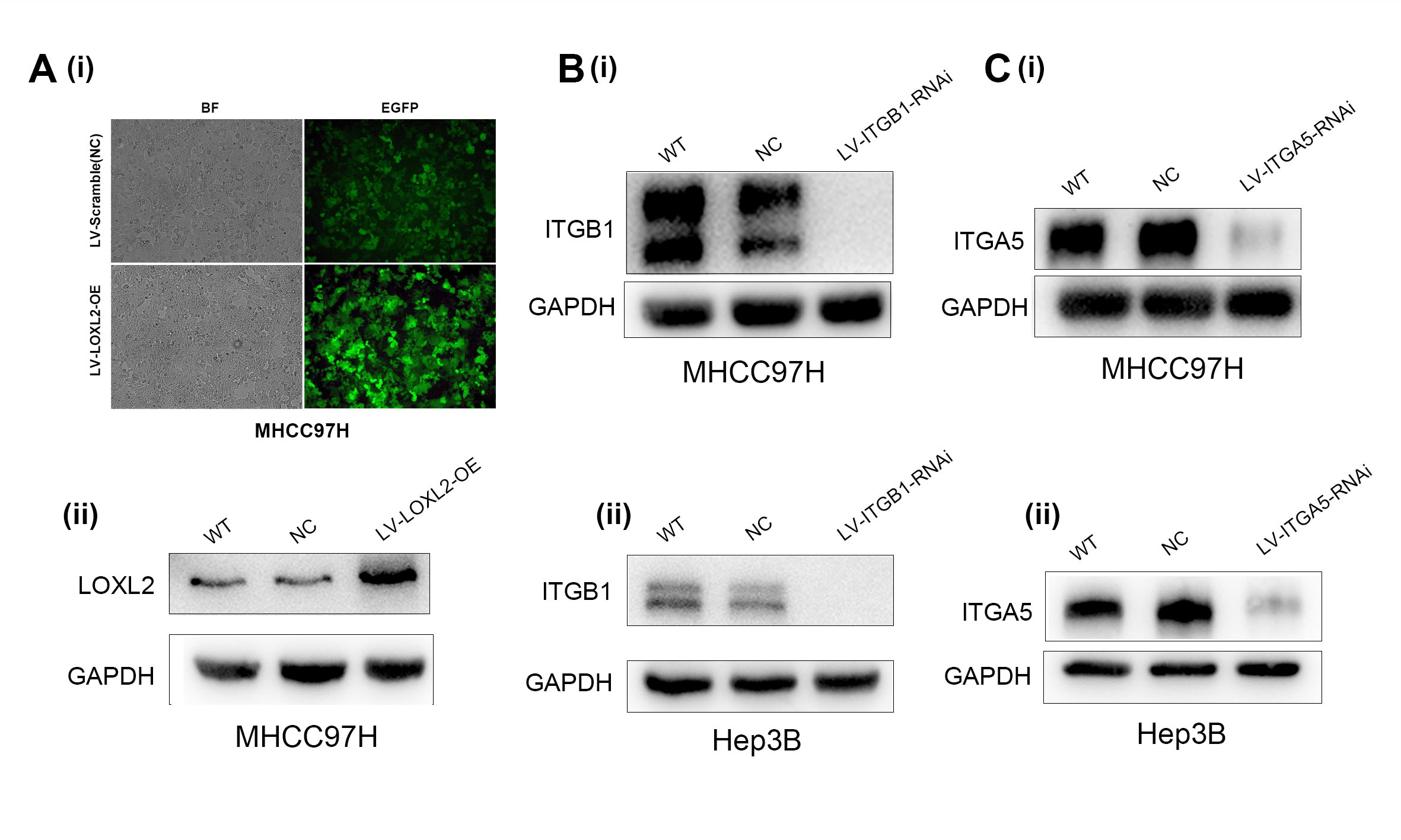

Supplement: Supplementary file 2 — Figure S1. Assessment of transfection efficiency. (A) (i) The transfected HCC cells with LV-LOXL2-OE. (ii) The expression level of LOXL2 in MHCC97H cells transfected with LV-LOXL2-OE. (B) The expression level of integrin β1 in HCC cells transfected with LV-ITGB1-RNAi. (C) The expression level of integrin α5 in HCC cells transfected with LV-ITGA5-RNAi. (JPG 336 kb) [file 13046_2018_761_MOESM2_ESM.jpg]
